# Long-term transmission of entangled photons from a single quantum dot over deployed fiber

**DOI:** 10.1038/s41598-019-40912-z

**Published:** 2019-03-11

**Authors:** Zi-Heng Xiang, Jan Huwer, R. Mark Stevenson, Joanna Skiba-Szymanska, Martin B. Ward, Ian Farrer, David A. Ritchie, Andrew J. Shields

**Affiliations:** 1 Toshiba Research Europe Limited, Cambridge Research Laboratory, 208 Science Park, Milton Road, Cambridge, CB4 0GZ UK; 2 Cavendish Laboratory, J. J. Thomson Avenue, Cambridge, CB3 0HD UK; 3 Present Address: Department of Electronic & Electrical Engineering, University of Sheffield, Sheffield, S1 3JD UK

## Abstract

Entangled light sources are considered as core technology for multiple quantum network architectures. Of particular interest are sources that are based on a single quantum system as these offer intrinsic security due to the sub-Poissonian nature of the photon emission process. This is important for applications in quantum communication where multi-pair emission generally compromises performance. A large variety of sources has been developed, but the generated photons remained far from being utilized in established standard fiber networks, mainly due to lack of compatibility with telecommunication wavelengths. In this regard, single semiconductor quantum dots are highly promising photon pair sources as they can be engineered for direct emission at telecom wavelengths. In this work we demonstrate the feasibility of this approach. We report a week-long transmission of polarization-entangled photons from a single InAs/GaAs quantum dot over a metropolitan network fiber. The photons are in the telecommunication O-band, favored for fiber optical communication. We employ a polarization stabilization system overcoming changes of birefringence introduced by 18.23 km of installed fiber. Stable transmission of polarization-encoded entanglement with a high fidelity of 91% is achieved, facilitating the operation of sub-Poissonian quantum light sources over existing fiber networks.

## Introduction

With the number of emerging quantum technologies rapidly increasing, the development of quantum networks is becoming more important than ever. Apart from special purpose networks suitable for secure key distribution^1–4^, more general architectures like a quantum internet^5,6^ are expected to unlock even greater potential for applications like cloud-based quantum computing^7,8^ or quantum sensing^9,10^. Apart from many other challenges, the most basic discipline in a general purpose quantum network is the generation and detection of entangled quantum bits (qubits). For true scalability not only in distance but also for widespread implementation, approaches are required that enable these two essential tasks in the most simple and robust way.

Since the early days, photon pair sources based on spontaneous processes like down conversion^11^ and more recently four-wave mixing^12^ were the most prominent choice for photonic entanglement generation. Over the years, the technology has evolved, enabling a large number of quantum-network related experiments. But due to their spontaneous nature, these sources can increase error rates in certain security-relevant applications caused by multi-pair emission^13^. Sub-Poissonian photon pair sources based on a single quantum emitter such as semiconductor quantum dots (QD) are a promising alternative, as they provide intrinsic security against photon number splitting attacks. Ongoing improvement of these sources is expected to reach a level which is approaching the performance of SPDC-based entangled photon sources in the near future^14^. Regarding photonic qubits, there exist two competing approaches for their implementation over optical fiber. Quantum states being encoded in the photon polarization are naturally favored for qubit generation in sources being based on single quantum emitters^15–19^, as they can directly interface with electronic states on optical dipole transitions. However, for long-distance transmission over optical fiber, polarization qubits are affected by random drifts in birefringence due to changing environmental conditions. In contrast, qubits encoded in the phase of subsequent time-bins are realized in a single polarization basis, protecting their information from birefringence-induced rotations and enabling simple stabilization schemes^20–23^. The major disadvantage is that stable interferometers are required for generation and detection, significantly increasing the complexity of the systems.

For certain scenarios, where multiple users are sharing the same quantum channel, the use of polarization encoding might be beneficial. The network provider could take care of the stabilization of birefringence, allowing the end user to operate with cheaper and less sensitive technology, important for scalable and wide-spread implementation. A number of experiments have been performed in the past, employing the transmission of polarization qubits over short specially installed non-telecom fiber without^24^ and with^25^ active stabilization. Efficient feedback systems for the continuous recovery of arbitrary polarization states operating at telecom wavelength have been demonstrated, enabling the stable transmission of polarization qubits from weak coherent sources over long fiber in a laboratory^26–28^ and from a down-conversion source over deployed fiber^29^. Here, we implement a similar polarization-control system to stabilize birefringence in 18 km of installed fiber across the city of Cambridge. We make use of a telecom-wavelength semiconductor quantum dot as photon source and show stable long-term operation of polarization-entangled photon transmission from a sub-Poissonian emitter over a standard telecommunication-fiber network link.

## Experiment

In recent years, effort has been put to push the emission wavelength of QDs to the standard telecommunication bands, enabling sub-Poissonian entangled photon-pair sources compatible with standard telecommunication infrastructure^30–33^. The InAs/GaAs quantum dot used in this work and our previous work^34^ is located in a PIN structure grown by molecular beam epitaxy with AlGaAs/GaAs stacked Bragg mirrors at the P type and N type layer in order to enhance photon collection. The ground state of a QD can be occupied with a maximum of two electron-hole pairs which decay in a cascade from the so-called biexciton level (XX) via the intermediate exciton level (X). The two subsequently emitted photons are maximally entangled in their polarization^15^ corresponding to the Bell state $|{{\rm{\Phi }}}^{+}\rangle =\frac{{\rm{1}}}{\sqrt{{\rm{2}}}}\,(|{{\rm{H}}}_{{\rm{X}}}{{\rm{H}}}_{{\rm{XX}}}\rangle +|{{\rm{V}}}_{{\rm{X}}}{{\rm{V}}}_{{\rm{XX}}}\rangle )$ with H and V denoting horizontal and vertical polarization respectively. A continuous wave laser at 1064 nm is used to optically excite the emitter at a temperature of 10 K, below the bandgap of GaAs, resulting in the so-called quasi-resonant injection of carriers. We apply a bias of −0.165 V to the diode, tuning the emission wavelength of XX photons to 1320.0 nm and X photons to 1329.4 nm.

**Figure 1:**
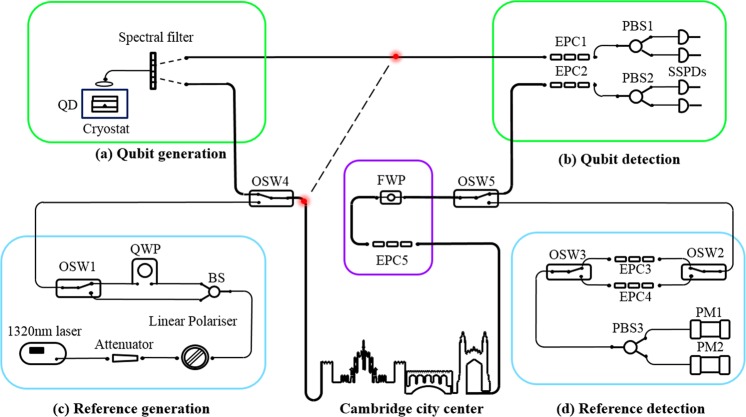
Experimental setup: The green boxes highlight the qubit generation (**a**) and detection (**b**) modules. The entangled photon pairs are emitted from a quantum dot (QD). Polarization correlations in different detection bases are measured with a fiber based polarization analyzing setup comprising electronic polarization controllers (EPC) 1 and 2, polarizing beam splitters (PBS) 1 and 2, and 4 superconducting single photon detectors (SSPD). The blue boxes highlight the birefringence stabilization system. Two polarization references are generated from a laser in (**c**) and detected in (**d**) in their respective detection bases using EPC 3 and 4, PBS 3 and power meters (PM 1, 2). Multiplexing of the two references and the bases is achieved using optical switches (OSW) 1 2 and 3. Detected polarization changes are compensated by applying feedback to a fiber wave plate (FWP) and EPC 5 in the purple square. OSW 4 and 5 are used for multiplexing and de-multiplexing the quantum channel and the reference channel over the field fiber. The spectral filter in (**a**) and the quarter wave plate (QWP) and linear polarizer in (**c**) are free-space optics, the rest of displayed components are all-fiber based.

Figure 1 illustrates the overall experimental setup. Polarization entangled photon pairs in the telecom O-band are generated from the quantum dot in (a). The light emitted from the device is coupled to single-mode fiber using a confocal microscope configuration, and passes through a free-space spectral filter to isolate entangled qubits into two separate single modes. One photon of a pair is transmitted over short optical fiber to a polarization analyzing setup directly in the lab, whereas the partner photon is sent over a loop-back fiber to the city center of Cambridge before detection back in the laboratory (b). Boxes (c) and (d) display the setup required for polarization stabilization over the deployed fiber.

The main difficulty in transmitting qubits encoded in polarization is to overcome polarization rotations induced by temporal variations of birefringence in installed fibers. The general approach is to inject polarization references which enables efficient detection and compensation of these variations when applying feedback to a set of polarization controllers. Over the network fiber, we observe a strong wavelength dependence of birefringence^35^. This results in a change of polarization states by 20 degrees on average on the Poincaré sphere for small variations in wavelength of 1 nm. Changes in environmental conditions cause a continuous variation of the birefringence^36^, resulting in polarization states of different wavelength evolving in a different random way. Therefore, wavelength-division multiplexing schemes^26^ could not be implemented for injection and retrieval of the references. We thus apply a time-division multiplexing scheme^29^, with both references and the quantum light at exactly the same wavelength.

For generation of the two references in Fig. 1(c) we split polarized laser light at 1320 nm in two separate spatial modes and use a free-space quarter wave plate (QWP) in one mode for a precise and long-term stable alignment of both polarizations along two orthogonal directions on the Poincaré sphere. In this configuration, the stabilization of both references efficiently locks arbitrary rotations of the sphere. An optical switch (OSW 1) is used to select either one of the two references. Two standard power meters in (d) are used to evaluate the projection *η* along the corresponding bases on the Poincaré sphere, controlled by electronic polarization controllers (EPC) 3 and 4 and polarizing beam splitter (PBS) 3:

1 $$\eta =({P}_{1}-{P}_{2})/({P}_{1}+{P}_{2})$$

where *P*_1_ and *P*_2_ denote the power values measured by each power meter. OSW 2 and 3 are used for fast and reliable switching between the two detection bases. Once a drop in *η* is detected for one of the references, EPC 5 and a fiber variable wave plate (FWP) in the purple box are used for applying rotations such that both references are recovered to their original state. Applying a voltage to the FWP results in a clean variable rotation of polarization states around a fixed axis on the Poincaré sphere. EPC 3 and 4 are set such that one detection basis coincides with the rotation axis of the FWP and the other one is oriented in plane of the rotation. Like this, cross-talk during the recovery of the references is minimized. As the FWP and all components for the reference detection in Fig. 1(d) are installed in a laboratory environment, the calibration of EPC 3 and 4 is done only once before the start of the experiment and typically stays stable over the course of a week. Both, EPC 5 and FWP are all-fiber based standard components featuring low insertion loss (<0.8 dB) and low-voltage operation.

**Figure 2:**
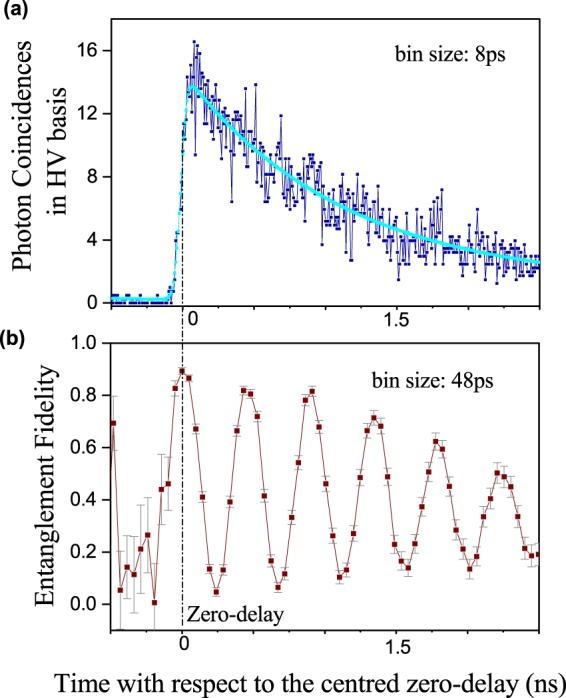
Photon correlations and entanglement fidelity from the QD emitter for a single experimental data set. (**a**) Normalized coincidences for co-polarized X and XX photons in the HV detection basis. The solid line is an empirical fit to extract the zero delay between the two photons of a pair. (**b**) Entanglement fidelity as a function of the delay between X and XX photon.

Photon entanglement is analysed by processing time-resolved correlations *c*_MN_ between measured X and XX photon arrival times for co- and cross-polarized states M and N in the three detection bases HV, DA (diagonal, anti-diagonal) and RL (right-, left-circular). These bases are initially calibrated using EPC 1 and 2 after injecting polarization references at the position of the QD, not displayed in Fig. 1. Similar to the setup for polarization reference detection, the components displayed in Fig. 1(b) are installed in a laboratory environment, resulting in a stable polarization alignment for typically more than a week. The fidelity to the maximally entangled Bell $|{{\rm{\Phi }}}^{+}\rangle$ state is calculated as^37^

2 $$F=\mathrm{(1}+{C}_{{\rm{HV}}}+{C}_{{\rm{DA}}}-{C}_{{\rm{RL}}})/4$$

with ${C}_{{\rm{MN}}}=({c}_{{\rm{MM}}}-{c}_{{\rm{MN}}})/({c}_{{\rm{MM}}}+{c}_{{\rm{MN}}})$ being the correlation contrast. Both, source and superconducting single photon detectors (Single Quantum) are running continuously during the measurements. The detection basis is switched every 10 min such that the fidelity can be evaluated for every 30 min of correlation data. An example for one set of data is shown in Fig. 2(b). Due to imperfections in the QD morphology, the fidelity follows a time-dependent oscillation caused by interference of two non-degenerate decay channels in the cascade introduced by the so-called fine structure splitting^38,39^. In order to extract the entanglement fidelity to $|{{\rm{\Phi }}}^{+}\rangle$, a post selection window is applied to each data set, isolating correlations in a 48 ps interval around the zero delay of the cascade. By doing this post-selection, approximately 3% of the detected photon pairs are used in the analysis. As the fraction of photons being entangled according to the exact time-evolving state is much larger^39^, active compensation schemes^40^ could be implemented in the future for increasing the fraction of pairs corresponding to a static entangled state. Changes in the ambient temperature result in a change of the effective optical length of the installed fiber, making an exact knowledge of the zero delay crucial in this analysis. As both photons of a pair are detected by the same single photon counting unit, no active synchronization is required. We extract the time-of flight for each 30 min chunk of data from a fit to correlations *c*_HH_ between X and XX photons measured in the HV polarization basis (Fig. 2(a)). Over this time scale the drift is typically negligible. The overall timing jitter of the photon detection system is 70 ps with a detector efficiency of 60% and correlation data is analyzed on a 48 ps timing grid.

## Results

**Figure 3:**
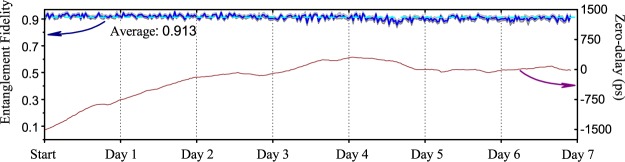
Entanglement fidelity and relative change of qubit transit time over the field fiber for 7 days of continuous operation. The mean fidelity is (91.3 ± 1.4)%. The gray shaded area indicates one standard deviation. Error bars for the delay values are negligible.

The entanglement fidelity has been continuously recorded for 7 days, with the XX photons being sent over the field fiber. Typical total photon detection rates were 320 kHz for X photons and 10 kHz for XX photons. The overall results are shown in Fig. 3. Displayed is the entanglement fidelity and the change in time-of-flight as a function of measurement time. A constantly high entanglement fidelity is achieved over the entire week, with an average value of (91.3 ± 1.4)%. For comparison, the entanglement fidelity measured without sending the photons over the loop-back link is (94.7 ± 1.7)%. The latter is mainly limited by imperfections of the fiber-based polarization detection system and re-excitation of the XX state from the intermediate X level, deteriorating the polarization correlation of subsequently emitted photons on the cascade. The increase of the uncorrelated-to-correlated coincidence ratio due to a much lower signal-to-background ratio when transmitting XX photons over the field fiber is the dominant effect responsible for the observed drop in fidelity. During the course of the week, rain has dropped and snow has fallen over Cambridge, with the temperature varying from −4 to 7 °C. The impact of these changing environmental conditions can be seen in the drift of the time-of-flight of photons of 1.82 ns over the first 4 days.

**Figure 4:**
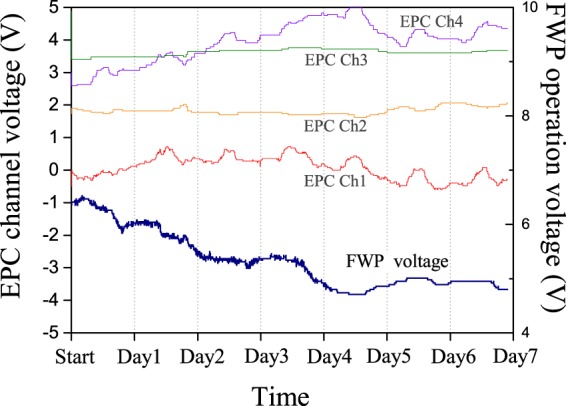
Operation voltage for EPC5 and FWP over the course of the measurement.

Figure 4 shows the voltages applied to the four channels of the EPC and the FWP as a result of the feedback generated to keep the birefringence stable. While there is almost no change in the voltages applied to channels 2 and 3 of the EPC, channels 1 and 4 are mainly dominated by oscillations corresponding to the day-night cycle of temperatures. This effect is most likely caused by birefringence induced by the sections of the installed fiber that are over ground and susceptible to short-term changes in temperature. In contrast, these oscillations are not visible in the voltage applied to the FWP, which seems to follow the drift of the time-of-flight instead. This can be explained by changes in birefringence that are induced by the much larger fraction of the fiber being installed under ground, which is mainly sensitive to the much slower change of the average outside temperature.

When using time-division multiplexing schemes, it is essential to achieve high duty cycles for qubit transmission. Over the course of the measurement, the polarization references were checked for 0.5 s alternatingly every 60 s, corresponding to a duty cycle of 99%. Once the projection value *η* dropped below a threshold of 98.5%, feedback for recovery was applied which took around 5 s on average, resulting in a duty cycle of 92%. Apart from the threshold-based check, the recovery system was activated every 11 minutes to maximize the alignment for both references. Thus, an overall duty cycle of 98% with polarization maintenance over 98.5% was realized for the qubit transmission. Since the optical switches used for multiplexing allow for switching speeds above 100 Hz it will be straight forward to adopt the system to much longer field fibers experiencing higher drift rates than the link available for this work.

Quantum light sources based on single quantum emitters are one of the most precious resources in a quantum network. As the brightness of these sources cannot be easily tuned like attenuated lasers, it is crucial that independent feedback systems add as little loss as possible. With this in mind, the system has been designed using standard fiber components with low insertion loss. The combined losses of optical switches OSW 4 and 5 used for multiplexing between quantum signal and references, and EPC 5 and FWP used for stabilization, add up to 3.49 dB, which can be further reduced by splicing. The installed loop back of standard telecom fiber has a total length of 18.23 km and a measured loss of 11.70 dB for the transmission of photons at 1320 nm.

## Conclusion

In summary, we have reported for the first time the long-term transmission of polarization qubits from a single quantum-dot emitter over 18.23 km of installed standard telecom fiber. The transmitted photons exhibit a constantly high entanglement fidelity of 91.3% with their partner photons measured locally, corresponding to a drop in fidelity by only 3.4% with respect to the source properties. The system has a low systematic loss and a high duty cycle that allows a high transmission efficiency of the qubits. The results demonstrate that quantum-dot emitters natively operating at telecom wavelength, combined with the deployment of entangled qubits over installed fiber, provide a reliable and stable technology which is highly competitive in terms of its low level of complexity, regarding qubit generation and robust detection schemes.

## Data Availability

The data generated during the current study are available from the corresponding author on reasonable request.
